# Wingless-Type MMTV Integration Site Family Member 5a Is a Key Secreted Islet Stellate Cell-Derived Product that Regulates Islet Function

**DOI:** 10.1155/2019/7870109

**Published:** 2019-04-11

**Authors:** Wei Xu, Jun Liang, H. F. Geng, Jun Lu, Rui Li, X. L. Wang, Qian Lv, Ying Liu, Jie Wang, X. K. Liu, Peter M. Jones, Zl Sun

**Affiliations:** ^1^Department of Endocrinology of Xuzhou Central Hospital, Xuzhou Institute of Medical Sciences, Affiliated Hospital of Southeast University, Xuzhou, Jiangsu, China; ^2^Department of Diabetes, School of Life Course Sciences, King's College London, Guy's Campus, London, UK; ^3^Department of Endocrinology, Zhongda Hospital, Institute of Diabetes, Medical School, Southeast University, Nanjing, China; ^4^Key Laboratory of Biotechnology on Medicinal Plants of Jiangsu Province, School of Life Science, Jiangsu Normal University, Xuzhou, China

## Abstract

**Background:**

Emerging evidence suggests that T2DM is attributable to the dysfunction of *β*-cells and the activation of islet stellate cells (ISCs). The wingless-type MMTV integration site family member 5a (Wnt5a)/frizzled 5 (Fzd5) signalling pathway might take part in this process. Our study is aimed at defining the status of ISCs during *β*-cell insulin secretion homeostasis by determining the role of the Wnt5a protein in the regulation of insulin production. We examined the effects of the status of ISCs on *β*-cell insulin secretion in normoglycemic db/m and hyperglycaemic db/db mice.

**Methods:**

iTRAQ protein screening and RNA interference were used to determine novel ISC-derived secretory products that may use other mechanisms to influence the function of islets.

**Results:**

We showed a significant reduction in insulin secretion by *β*-cells in vitro when they were cocultured with db/db ISCs compared to when they were cocultured with ISCs isolated from normoglycemic db/m mice; in addition, both Wnt5a and its receptor Fzd5 were more highly expressed by quiescent ISCs than by activated db/db ISCs. Treatment with exogenous Wnt5a increased the secretion of insulin in association with the deactivation of ISCs.

**Conclusion:**

Our observations revealed that the Wnt5a protein is a key effector of ISC-mediated improvement in islet function.

## 1. Introduction

T2DM is one of the most common metabolic disorders characterised by resistance to the action of insulin (IR), increased rates of endogenous glucose production, reduced insulin secretion, and *β*-cell dysfunction [1]. Continuous insulin resistance will progress to T2DM when *β*-cells are unable to secrete adequate insulin to compensate for the decreased insulin sensitivity, which is largely due to insulin secretory dysfunction of *β*-cells [2, 3]. Understanding the mechanisms underlying insulin synthesis, secretion, and regulation will undoubtedly be important for the prevention and treatment of diabetes. Emerging evidence suggests that islet dysfunction and the activation of pancreatic stellate cells (PSCs) play an important role in the pathogenesis of diabetes [4–9]; when activated by a range of high-glucose, inflammation environmental stimuli that are also associated with T2DM, PSC activation produces an excessive extracellular matrix (ECM) involved in islet fibrosis leading to diabetes [10, 11].

Various cells within islets can influence insulin synthesis and secretion via hormones, and other substances, such as somatostatin (*δ* cells) [12, 13], glucagon (*α* cells) [14], and pancreatic polypeptide (PP cells) [15], also regulate insulin secretion. The means of physiological communication among these cells inside the islets has not yet been fully clarified.

Previous studies demonstrated a type of stellate cell located in the inner part of the islet (ISC), which is similar in appearance to pancreatic stellate cells but distinctly different. Our recent in vitro study confirmed that ISCs are activated in T2DM, and treatment with Reg1 suppressed the activation of diabetic ISCs [16]. However, the roles played by ISCs during *β*-cell insulin secretion homeostasis have not been fully elucidated. Understanding the role of stellate cells in regulating islet function can help us classify the pathogenesis of diabetes and find new targets for improving the treatment of diabetes.

Wnt5a, a member of the Wnt family of secreted glycoproteins, is a prototypical Wnt of the *β*-catenin-independent branch that plays important roles in embryonic development, postnatal tissue homeostasis, and pathological disorders throughout the lifespan of an organism [17, 18]. Wnt5a signalling is associated with diverse pathogenic diseases, such as inflammatory diseases, metabolic disorders, and cancer [19]. Wnt5a/Fzd5 signalling is involved in proper islet formation and insulin-mediated cell migration during progress of vertebrate pancreatic development [20, 21]. Interestingly, our in vitro study showed that expression profiles of Wnt5a and Fzd5 could be found in db/m ISCs and that Wnt5a increased the secretion of insulin. However, the underlying mechanisms of the associations among ISCs/Wnt5a/*β*-cells are not clear. Because quiescent ISCs serve a physiological function in islets, and the Wnt5a protein can maintain *β*-cell insulin secretion homeostasis, maintenance of the viability of quiescent ISCs is a desirable outcome of therapeutic strategies for T2DM.

The individual pathophysiological roles of ISCs have been performed extensively, but we have little understanding of ISCs and Wnt5a in the regulation of insulin secretion. Our study is aimed at defining the roles played by ISCs during *β*-cell insulin secretion homeostasis and imbalance and investigating the regulation of insulin production by the Wnt5a protein.

## 2. Materials and Methods

### 2.1. Animals

8-12-week-old male db/db mice and sex-matched male db/m mice were purchased from the Model Animal Research Center of Nanjing University (Nanjing, China). This study protocol was reviewed by the Animal Care and Use Committee according to institutional guidelines and national animal welfare.

### 2.2. Isolation and Culture of Mouse Islet Stellate Cells

Islets and ISCs were isolated from hyperglycaemic db/db and normoglycemic db/m mice as described previously [16]. Isolated mouse islets were divided into groups of 50 for culture alone or with exogenous Wnt5a (0.05 ng/ml) (Wnt5a; R&D Systems, UK) for 48 hours unless otherwise specified.

### 2.3. Measurement of Wnt5a Expression and Secretion

To assess the expression of Wnt5a in ISC lysates and to determine whether Wnt5a was released into culture media, ISCs were seeded into Nunclon™ 35 mm Petri dishes. After 72 hours, ISCs from each Petri dish were trypsinised, resuspended, and supplemented with complete ULTRA mini protease inhibitors and sonicated. The ISC-conditioned media (CM) was also collected and concentrated. The control samples were composed of the ISC culture media alone, which was also concentrated ×12. Wnt5a was measured in the ISC lysates and CM using iTRAQ protein screening.

### 2.4. Immunohistochemistry

To confirm the protein expression of Wnt5a and Fzd5 in pancreases, immunohistochemistry was performed as described previously [16]. The primary antibodies used were as follows: Wnt5a (1 : 200, Abcam, UK) and Fzd5 (1 : 200, Abcam, UK).

### 2.5. Apoptosis Assay

The level of apoptosis in ISCs was performed by A Caspase-Glo 3/7 (Caspase-Glo®, Promega) apoptosis kit as described previously [22].

### 2.6. Viability Assay

The level of viability in cells was performed by a WST-8 assay kit (Sigma, USA) [16].

### 2.7. Western Blotting Analysis

ISCs were cultured alone or in the presence of Wnt5a or shRNA for 48 hours. After 48 hours, Western blotting was performed [16], and the primary antibodies used were as follows: Wnt5a (1 : 3000, Abcam, UK), Fzd5 (1 : 3000, Abcam, UK), and *β*-actin (1 : 2000, Sigma, USA).

### 2.8. Islet Secretory Function In Vitro

Static incubation of islets was performed for assessing insulin secretion levels in vitro. Islets were preincubated for 2 hours in RPMI containing 2 mmol/l glucose. Each group of three islets was subsequently transferred into 1.5 ml Eppendorf tubes and incubated at 37°C in a buffer containing 2 mmol/l CaCl_2_, 0.5 mg/ml BSA, and either 2 or 20 mmol/l glucose. After 1 hour, the incubation medium was retained and stored at -20°C until they were assayed for their insulin content using an insulin ELISA kit. Islet insulin content was assessed as described previously [23, 24].

Insulin secretion in vitro was also determined in perifusion incubations of isolated islets. All the work is done in a room at 37 degrees Celsius. The perifusion medium (KRBB 2.8 mmol/l glucose, 16.7 mmol/l glucose) was maintained at 37 degrees Celsius in a water bath. Groups of size-matched 50 islets were placed in each column. The columns were gently immersed in vertical position, and all columns were perifused in parallel with 2.8 mmol/l glucose at a flow rate of 0.5 ml/min. After 60 min, the islets were stimulated at a flow rate of 0.5 ml/min in the continuous presence of a high concentration of 16.7 mmol/l glucose. Samples of the incubation medium were collected every 20 seconds until 2 min, every 1 min until 5 min, and every 5 min until 30 min. Samples were immediately taken and stored at -20 degrees Celsius until further analysis. Insulin content was assessed as described previously [23, 24].

### 2.9. shRNA Preparation and Targeting Gene Knockdown

We performed a BLAST search to confirm that the shRNA constructs targeted only mouse Wnt5a (+/+) and Fzd5 (-/-). The lentiviruses containing the targeted gene shRNA were synthesized, and ISC cell transfection was performed according to the manufacturer's instructions. Specific shRNAs and control shRNA were synthesized and provided by GeneChem company (Shanghai, China).

### 2.10. Statistical Analysis

The results were shown as means ± SEM for quantitative date. The statistical multiple comparisons ware determined using Bonferroni's *T* test with SAS, and differences were considered significant when *P* < 0.05.

## 3. Results

### 3.1. The Effects of Different ISC Profiles on Islet Insulin Secretory Function

**Figure 1:**
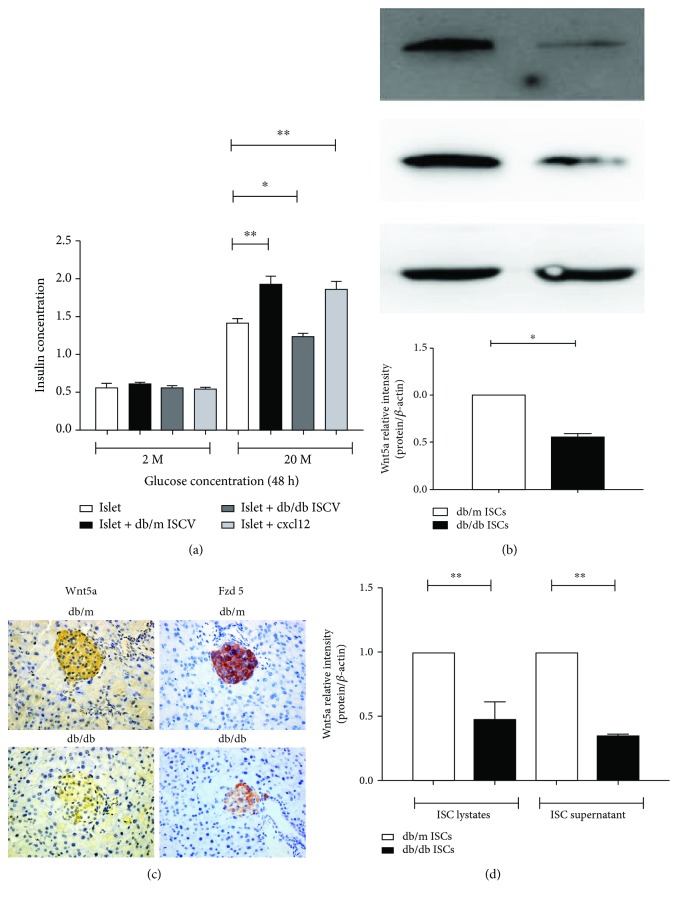
The effects of different ISC profiles on islet insulin secretory function and Wnt5a content and release by ISCs. (a) Compared with coculture with ISCs isolated from normoglycemic db/m mice, coculture of islets with ISC isolation from db/db mice significantly reduced insulin secretion in vitro. All data were expressed as mean ± SE (*n* = 3), ^∗^*P* < 0.05 and ^∗∗^*P* < 0.01, normoglycemic db/m ISCs vs. hyperglycaemic db/db ISCs. (b) Protein levels of Wnt5a were increased in control db/m ISCs than db/db ISCs. All data were expressed as means ± SE (*n* = 3), ^∗^*P* < 0.05 and ^∗∗^*P* < 0.01, normoglycemic db/m ISCs compared with hyperglycaemic db/db ISCs. (c) Wax-embedded sections of db/m and db/db mouse pancreases showing the expression of Wnt5a and Frizzled 5 as revealed by immunohistochemistry. Scale bar = 50 *μ*m. (d) Protein levels of Wnt5a were increased in db/m ISCs lysates and supernatant than in the db/db ISCs. All data were expressed as means ± SE (*n* = 3), ^∗^*P* < 0.05 and ^∗∗^*P* < 0.01, normoglycemic db/m ISCs compared with hyperglycaemic db/db ISCs.

To investigate the effects of different ISC profiles on islet insulin secretion, we treated islets with db/m and db/db ISCs; compared with islets cocultured with ISCs from db/m mice, islets cocultured with ISCs from hyperglycaemic db/db mice had significantly decreased insulin secretion in vitro (Figure 1(a)). Together, these data demonstrate a potential role played by quiescent ISCs in regulating islet function.

### 3.2. Wnt5a Content and Release by ISCs

To assess the levels of Wnt5a expression, as shown in Figure 1(b), western blotting analysis revealed that the expression of Wnt5 and Fzd5 in the ISCs isolated from db/db mice was significantly reduced compared with those of normoglycemic db/m mice. We also used iTRAQ to quantify Wnt5a in lysates from ISCs and in ISC-CM. ISC extracts contained 523 ± 5 pg Wnt5a/100,000 cells (mean ± SEM, *n* = 3), and Wnt5a protein expressed by ISCs was released into the medium approximately 30% (165 ± 12 pg/well, *n* = 7). Control samples of ISC-CM maintained for 3 days without ISCs contained negligible amounts of Wnt5a immunoreactivity (<0.1% of ISC-CM) (Figure 1(d)). These observations show that ISCs synthesize and release Wnt5a, consistent with it mediating the influence of ISCs on islet function.

### 3.3. Expression of Wnt5a and Frizzled5 in the Pancreas and Islets

We use immunohistochemistry to examine the expression of Wnt5a and Fzd5 in pancreatic sections, as shown in Figure 1(c). Lightly stained Wnt5a+ and Fzd5+ cells were shown in the pancreas from hyperglycaemic db/db mice. Wnt5a and Fzd5 expression in the pancreatic tissue of normoglycemic db/m mice is much higher. Together, the above data shows that Wnt5a and frizzled-5 expression in pancreatic tissue is significantly higher from normoglycemic db/m mice than in those from diabetic mice.

### 3.4. Wnt5a Increases Insulin Secretion and Inhibits the Activation of ISCs

**Figure 2:**
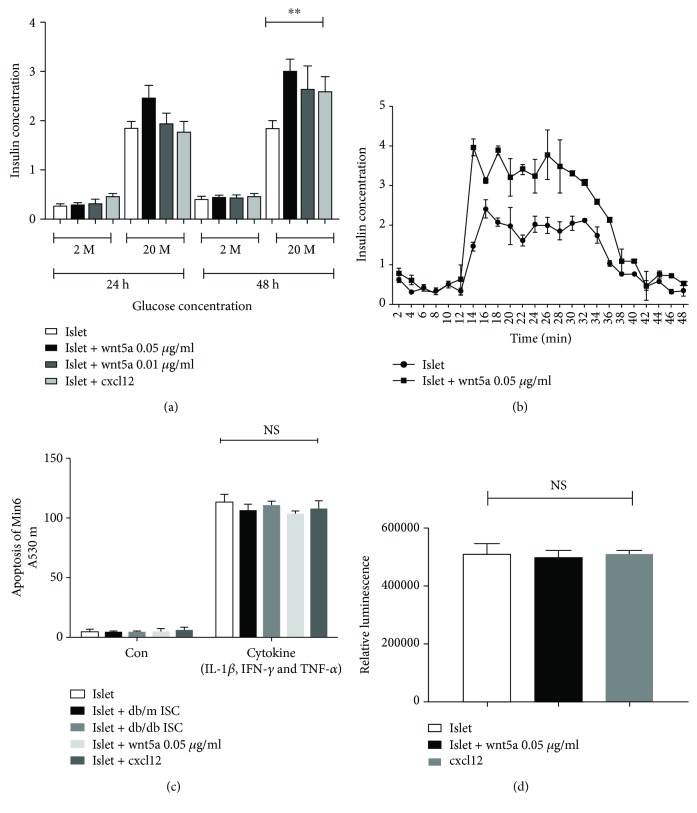
Control db/m ISCs and Wnt5a increase insulin secretion with no change in cytokine-induced apoptosis and viability. (a) The in vitro insulin was significantly increased by coculture with 0.05 *μ*g/ml rhWnt5a protein for 48 hours compared to coculture with the control (static). All data were expressed as means ± SE (*n* = 3), ^∗^*P* < 0.05 and ^∗∗^*P* < 0.01, db/m islets vs. db/m islets +0.05 *μ*g/ml Wnt5a. (b) The in vitro insulin secretion was significantly increased by coculture with 0.05 *μ*g/ml rhWnt5a protein for 48 hours compared to coculture with the control (perifusion). All data were expressed as means ± SE (*n* = 3), ^∗^*P* < 0.05 and ^∗∗^*P* < 0.01, db/m islets vs. db/m islets +0.05 *μ*g/ml Wnt5a. (c) Assay of apoptosis in islets from the cytokine-induced and control groups. There was no change in the level of apoptosis. (d) Assay of viability in islets from the cytokine-induced and control groups. There was no change in the level of viability.

The effects of exogenous Wnt5a on glucose-stimulated insulin secretion from static incubations of isolated islets were investigated, as shown in Figure 2(a). Treatment of exogenous Wnt5a had no effect on basal (2 mmol/l glucose) insulin secretion from isles which had been pretreated for 48 h, but significantly increased glucose-stimulated (20 mmol/l) insulin secretion. This effect was reproducible, and Figure 2(b) shows that the sufficient mimic results from perifusion incubations of isolated islets in which exogenous Wnt5a significantly enhanced glucose-induced insulin secretion. Figures 2(c) and 2(d) show that preculture for 48 h in the presence of 0.05 *μ*g/ml exogenous Wnt5a had no effect on apoptosis and viability of islets.

**Figure 3:**
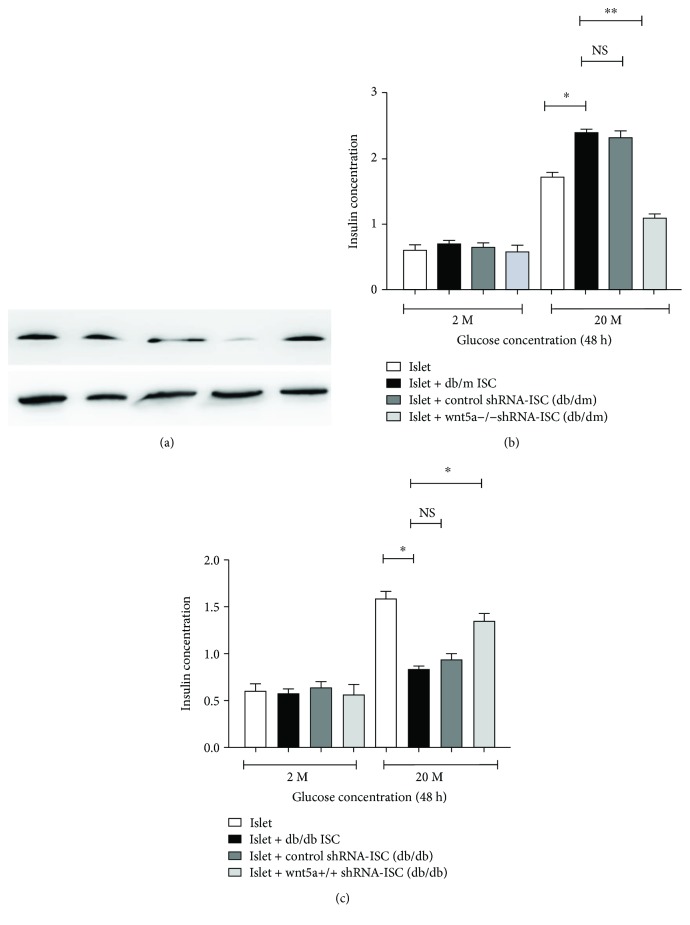
Wnt5a protein is a key modulator of ISC-mediated regulation of islet function. (a) A significant change in the mRNA expression of Wnt5a at 72 hours was caused by transfection of ISCs with shRNAs directly targeting Wnt5a when compared to control. All data were expressed as means ± SE (*n* = 3), ^∗^*P* < 0.05 and ^∗∗^*P* < 0.01, shRNA Wnt5a-ISCs vs. ISCs transfected with control nontargeting shRNAs. (b) Islets cocultured with Wnt5a-/-db/m ISCs had significantly reduced insulin secretion compared with control. All data were expressed as means ± SE (*n* = 3), ^∗^*P* < 0.05 and ^∗∗^*P* < 0.01, shRNA Wnt5a-/--db/m ISCs vs. db/m ISCs transfected with control nontargeting shRNAs. (c) The shRNA-induced upregulation of Wnt5a expression in hyperglycaemic db/db ISCs increased the insulin secretion compared with the control. All data were expressed as means ± SE (*n* = 3), ^∗^*P* < 0.05 and ^∗∗^*P* < 0.01, shRNA Wnt5a+/+-db/db ISCs vs. db/db ISCs transfected with control nontargeting shRNAs.

**Figure 4:**
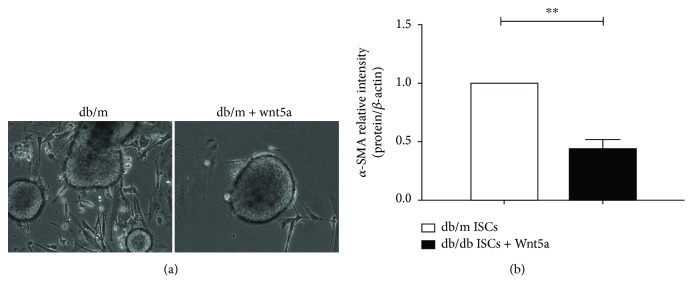
Wnt5a inhibits the activation of ISCs. (a) The rates of ISC outgrowth from islets treated with Wnt5a were demonstrated by light microscopy micrograph demonstration. Scale bar = 50 *μ*m. (b) Western blotting of normoglycemic db/m ISCs treated with Wnt5a via the *α*-SMA antibody. Data are expressed as means ± SE (*n* = 3), ^∗^*P* < 0.05 and ^∗∗^*P* < 0.01.

To determine the effects of Wnt5a on insulin secretion, shRNA-mediated knockdown (db/m ISCs) and upregulation (db/db ISCs) of Wnt5a were used. Immunoblot analysis confirmed that the expression level of the Wnt5a protein was substantially changed at 72 hours posttransfection (Figure 3(a)). Compared with transfection with control nontargeting shRNAs, transfection with shRNAs directly targeting Wnt5a caused a significant change in the protein expression of Wnt5a at 72 hours (Figure 3(a)). Insulin secretion by islets after coculture with Wnt5a-/-db/m ISCs was significantly reduced compared to treatment with control nontargeting shRNAs. Conversely, upregulation of Wnt5a in db/db ISCs after using shRNA induced insulin secretion, as evaluated using the same parameters (Figures 3(b) and 3(c)). Meanwhile, we investigated the rate of ISC outgrowth and the expression of *α*-SMA after coculture with Wnt5a. The level of Wnt5a decreased the rate of ISC outgrowth from normoglycemic db/m islets, and the expression of *α*-SMA was decreased when compared to expression in the control (Figures 4(a) and 4(b)). Together, these results demonstrate that quiescent ISCs play a potential role in regulating islet function and that the Wnt5a protein is a key modulator of ISC-mediated improvements in islet function.

## 4. Discussion

*β*-cell failure is central to the development and progression of T2DM [25]. Previous studies have confirmed that *β*-cell failure is associated with activation of stellate cells [26, 27].

In these studies, to extend these observations we have focused our research to show that interactions between ISCs regulate *β*-cell insulin secretion in vitro. The results demonstrated a significant decrease in insulin secretion in vitro by *β*-cells cocultured with ISCs isolated from db/db mice compared to *β*-cells treated with ISCs isolated from normoglycemic db/m mice. These observations are consistent with the activation of ISCs in response to the diabetic environment, leading to decreases in ISC-derived secretory products, which may influence the function of islet. Understanding the mechanisms leading to the quiescent state of ISCs may play an important role in regulating islet function, and activation of stellate cells in diabetic environment can serve as a target for the prevention of diabetes.

Our studies identified the differential expression of proteins, such as Reg1, annexin A1, Cxcl12, and Wnt5a, in ISCs isolated from db/db and db/m mice and GK and Wistar rat through iTRAQ protein screening. Subsequent research demonstrated that Reg1 can decrease the activation of ISCs [16]. In this study, we have identified the ISC/Wnt5a/*β*-cell system as one mechanism which influences the state of ISC and insulin secretion. Wnt5a, a member of the Wnt family of secreted glycoproteins, is a prototypical Wnt of the *β*-catenin-independent branch that plays important roles in the processes governing embryonic development and pathological disorders throughout the lifespan of an organism [28]. Wnt5a signalling has been linked to several human pathologies, such as inflammation, cancer, and fibrosis [29, 30]. Wnt5a/Fzd5 signalling plays a role in proper insulin-induced islet formation and cell migration during vertebrate pancreatic development [20, 21]. Plasma levels of secreted frizzled-related protein 5 (Sfrp5), known to be a secreted antagonist that binds to the Wnt5a protein, were found to be elevated in patients with T2DM and can play a role in influencing lipid metabolism, inflammation, and T2DM [31]. Interestingly, the level of circulating Sfrp5 was significantly lower in both individuals with impaired glucose intolerance and those with newly diagnosed T2DM than in individuals with normal glucose tolerance [32].

Our recent clinical study showed that Wnt5a levels were significantly downregulated in patients with newly diagnosed T2DM and gradually increased in long-term patients with diabetes with kidney disease. Research on the connections between the Wnt5a protein, Sfrp5, and diabetes is mainly concentrated on adipose tissue, inflammation, and IR. However, the associations among ISCs, the Wnt5a protein, and *β*-cell function have not been studied in T2DM.

In our study, Wnt5a and Fzd5 are expressed in normoglycemic db/m ISCs at a relatively high expression. However, the level of both molecules is significantly decreased in ISCs isolated from hyperglycaemic db/db mice, demonstrating that ISCs synthesize and release Wnt5a, which is consistent with its mediation of the influence of ISCs on islet function. The coculture of islets with db/db ISCs with shRNA-mediated upregulation of endogenous Wnt5a increased insulin secretion. Conversely, downregulation of Wnt5a expression in normoglycemic db/m ISCs by shRNA decreased islet insulin secretion, demonstrating that the increased insulin secretion by hyperglycaemic db/db ISCs was caused by the regulation of Wnt5a expression. Thus, a hyperglycaemic, inflammatory prediabetic environment leads to the downregulation of Wnt5a and then increase the activation of ISCs. It is important to prevent the ISCs from remaining in the quiescent state to activation, which is involved in regulating islet function.

Although these results clearly supported our hypothesis that ISCs are important for the maintenance of *β*-cell insulin secretion in a physiological environment, the effect of Wnt5a on *β*-cell insulin signalling has yet to be observed; biochip technology and transgenic models could be used to elucidate the meaning of this process in the pathogenesis of T2DM.

## 5. Conclusion

In conclusion, we have acknowledged a type of stellate cell located inner part of the islet that is similar in appearance to pancreatic stellate cells but distinctly different and have demonstrated that this population of quiescent ISCs regulates insulin secretion by *β*-cells through the Wnt5a protein. Quiescent ISCs will to be active in chronic exposure to a diabetic environment transforming them into a fibrotic phenotype, with a consequent reduction in the level of Wnt5a, suggesting that these cells participate in the regulation of insulin secretion and that the dysfunction of these cells leads to the pathogenesis of T2DM. These studies demonstrate that quiescent ISCs have a physiological function in islets and that the Wnt5a protein can maintain *β*-cell insulin secretion homeostasis, which is a key effector of ISC-mediated improvements in islet function. We demonstrate the specific mechanisms that novel ISC-derived secretory products influence islet function and hoped that these proteins could be applied to the pretreatment of islet transplantation and thus improve transplantation outcomes.

## Data Availability

The data used to support the findings of this study are available from the corresponding author upon request.

## Abbreviations

T2DM: Type 2 diabetes mellitus
ISCs: Islet stellate cells
Wnt5a: Wingless-type MMTV integration site family member 5a
Fzd5: Frizzled-5
IR: Insulin resistance
PSCs: Pancreatic stellate cells
ECM: Extracellular matrix
*α*-SMA: *α*-Smooth muscle actin
GFAP: Glial fibrillary acidic protein
BSA: Bovine serum albumin
Sfrp5: Secreted frizzled-related protein 5.
