# Corrigendum to “Wingless-Type MMTV Integration Site Family Member 5a Is a Key Secreted Islet Stellate Cell-Derived Product that Regulates Islet Function”

**DOI:** 10.1155/2019/6762534

**Published:** 2019-10-23

**Authors:** Wei Xu, Jun Liang, H. F. Geng, Jun Lu, Rui Li, X. L. Wang, Qian Lv, Ying Liu, Jie Wang, X. K. Liu, Peter M. Jones, Zl Sun

**Affiliations:** ^1^Department of Endocrinology of Xuzhou Central Hospital, Xuzhou Institute of Medical Sciences, Affiliated Hospital of Southeast University, Xuzhou, Jiangsu, China; ^2^Department of Diabetes, School of Life Course Sciences, King's College London, Guy's Campus, London, UK; ^3^Department of Endocrinology, Zhongda Hospital, Institute of Diabetes, Medical School, Southeast University, Nanjing, China; ^4^Key Laboratory of Biotechnology on Medicinal Plants of Jiangsu Province, School of Life Science, Jiangsu Normal University, Xuzhou, China

In the article titled “Wingless-Type MMTV Integration Site Family Member 5a Is a Key Secreted Islet Stellate Cell-Derived Product that Regulates Islet Function” [1], the keys of Figures 1(b) and 2(a) were missing. The correct Figures 1 and 2 are shown below.

## Figures and Tables

**Figure 1:**
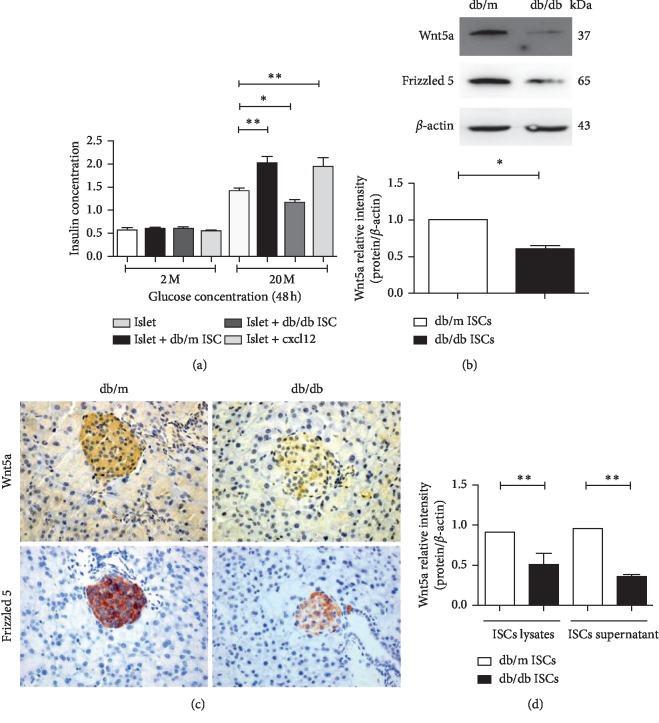
The effects of different ISC profiles on islet insulin secretory function and Wnt5a content and release by ISCs. (a) Compared with coculture with ISCs isolated from normoglycemic db/m mice, coculture of islets with ISCs isolation from db/db mice significantly reduced insulin secretion in vitro. All data were expressed as the mean ± SE (*n* = 3); ^*∗*^*P* < 0.05; ^*∗∗*^*P* < 0.01; normoglycemic db/m ISCs vs hyperglycaemic db/db ISCs. (b) Protein levels of Wnt5a were increased in control db/m ISCs than db/db ISCs. All data were expressed as the means ± SE (*n* = 3); ^*∗*^*P* < 0.05; ^*∗∗*^*P* < 0.01; normoglycemic db/m ISCs compared with hyperglycaemic db/db ISCs. (c) Wax-embedded sections of db/m and db/db mouse pancreases showing the expression of Wnt5a and Frizzled 5 as revealed by immunohistochemistry. Scale bar = 50 *μ*m. (d) Protein levels of Wnt5a were increased in db/m ISCs lysates and supernatant than in the db/db ISCs. All data were expressed as the means ± SE (*n* = 3); ^*∗*^*P* < 0.05; ^*∗∗*^*P* < 0.01; normoglycemic db/m ISCs compared with hyperglycaemic db/db ISCs.

**Figure 2:**
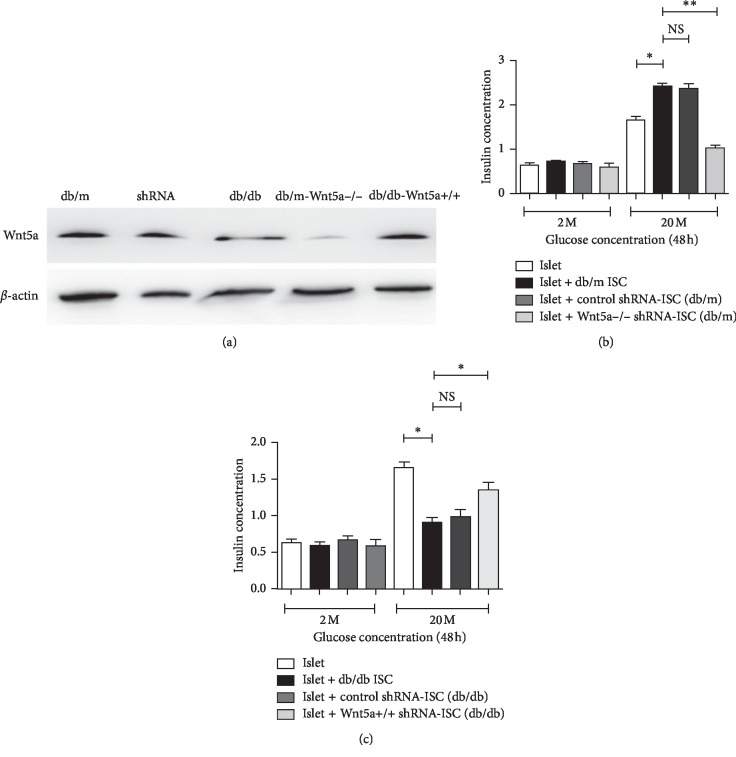
Wnt5a protein is a key modulator of ISC-mediated regulation of islet function. (a) A significant change in the mRNA expression of Wnt5a at 72 hours was caused by transfection of ISCs with shRNAs directly targeting Wnt5a when compared to control. All data were expressed as the means ± SE (*n* = 3); ^*∗*^*P* < 0.05; ^*∗∗*^*P* < 0.01; shRNA Wnt5a-ISCs vs ISCs transfected with control nontargeting shRNAs. (b) Islets cocultured with Wnt5a−/− db/m ISCs had significantly reduced insulin secretion compared with control. All data were expressed as the means ± SE (*n* = 3); ^*∗*^*P* < 0.05; ^*∗∗*^*P* < 0.01; shRNA Wnt5a−/− db/m ISCs vs db/m ISCs transfected with control nontargeting shRNAs. (c) The shRNA-induced upregulation of Wnt5a expression in hyperglycaemic db/db ISCs increased the insulin secretion compared with the control. All data were expressed as the means ± SE (*n* = 3); ^*∗*^*P* < 0.05; ^*∗∗*^*P* < 0.01; shRNA Wnt5a+/+ db/db ISCs vs db/db ISCs transfected with control nontargeting shRNAs.
